# Enhanced Dopamine Transmission and Hyperactivity in the Dopamine Transporter Heterozygous Mice Lacking the D3 Dopamine Receptor

**DOI:** 10.3390/ijms21218216

**Published:** 2020-11-03

**Authors:** Tatyana D. Sotnikova, Evgeniya V. Efimova, Raul R. Gainetdinov

**Affiliations:** 1 Institute of Translational Biomedicine, St. Petersburg State University, 199034 St. Petersburg, Russia; tdsotnikova@gmail.com (T.D.S.); e.v.efimova@mail.ru (E.V.E.); 2 St. Petersburg University Hospital, St. Petersburg State University, 199034 St. Petersburg, Russia

**Keywords:** dopamine transporter, dopamine receptor, transgenic animals, hyperlocomotion, thermoregulation

## Abstract

Dopamine transporter knockout (DATk) mice are known to demonstrate profound hyperactivity concurrent with elevated (5-fold) extracellular dopamine in the basal ganglia. At the same time, heterozygous DAT mice (DATh) demonstrate a 2-fold increase in dopamine levels yet only a marginal elevation in locomotor activity level. Another model of dopaminergic hyperactivity is the D3 dopamine receptor knockout (D3k) mice, which present only a modest hyperactivity phenotype, predominately manifested as stereotypical behaviors. In the D3k mice, the hyperactivity is also correlated with elevated extracellular dopamine levels (2-fold) in the basal ganglia. Cross-breeding was used to evaluate the functional consequences of the deletion of both genes. In the heterozygous DAT mice, inactivation of the D3R gene (DATh/D3k) resulted in significant hyperactivity and further elevation of striatal extracellular dopamine above levels observed in respective single mutant mice. The decreased weight of DATk mice was evident regardless of the D3 dopamine receptor genotype. In contrast, measures of thermoregulation revealed that the marked hypothermia of DATk mice (−2 °C) was reversed in double knockout mice. Thus, the extracellular dopamine levels elevated by prolonging uptake could be elevated even further by eliminating the D3 receptor. These data also suggest that the hypothermia observed in DATk mice may be mediated through D3 receptors.

## 1. Introduction

The dopamine system is involved in the regulation of many vital physiological functions, including locomotor activity, hormonal status, and thermoregulation [1]. Extracellular dopamine levels in the basal ganglia can be regulated by several mechanisms. The major one is the rapid reuptake of dopamine from the synaptic cleft by the dopamine transporter (DAT). Lack of DAT in knockout animals results in remarkable changes in behavior, physiology, and neurochemistry [2,3]. DAT knockout (DATk) mice and rats have a 5–7 fold elevated level of striatal extracellular dopamine, with about 15–20-fold decreased level of total tissue content of dopamine [2,3,4]. DATk mice show dramatically increased locomotor activity in a novel environment that can be attenuated by the administration of amphetamine [5]. During postnatal development, DATk mice show decreased bodyweight that also manifested in adult animals [2,6]. It was also shown that DATk animals have a lower body temperature during the daytime [7].

On the cellular level, the persistent hyperdopaminergia in DATk mice results in changes in dopamine receptor function. It has been shown that the gene expression of both D1 and D2 dopamine receptors is decreased, whereas the expression of the D3 dopamine receptor gene in the striatum is increased in DATk mice [8]. D3 dopamine receptors belong to the D2-like dopamine receptor subclass known to act via Gi cascade and to be present both on the presynaptic membrane and postsynaptic structures. D2-class receptors are generally thought to be acting as autoreceptors [9] that represent another major feedback mechanism for regulation extracellular dopamine levels.

To study the function of dopamine D3 receptors, mice lacking these receptors were created [10,11]. The autoreceptor function of the D3 receptor was demonstrated by detecting significant alterations in the striatal dopamine system of D3 knockout (D3k) mice. D3k mice have higher levels of extracellular dopamine as measured by microdialysis, however, with an unaltered firing rate of dopamine neurons [12,13]. Significant behavioral changes have been shown in D3k mice. D3k animals exhibited locomotor hyperactivity [10]. Apart from increased locomotor activity, D3k animals showed increased stereotypy [13] resulting in a higher basal grooming rate [14]. Furthermore, D3k mice exhibited higher anxiety levels in the open field test and depressive-like behaviors in Porsolt forced swimming test [15].

In this work, we created double knockout mice deficient in both DAT and D3 receptors to further study the interaction of these two mechanisms of extracellular dopamine regulation and their influence on neurochemistry, behavior, and major physiological parameters.

## 2. Results

As DAT and D3 genes are located in different chromosomes [1,2,16], mice heterozygous for respective genes [13,16] were bred to receive double heterozygous mice, which were further cross-bred to receive expected 9 genotypes according to mendelian distribution. All genotypes appeared to be viable at birth. However, double-mutant mice which were knockout mice for the DAT gene (DATk) and heterozygous for D3 gene (D3h) demonstrated excessive grooming so severe that it resulted in major self-injuring of the skin of paws and back (visual observations). Therefore, according to bioethical rules, the majority of these animals had to be euthanized and we could not obtain sufficient animals to evaluate their parameters. Double knockout mice (DATk/D3k) also demonstrated excessive grooming and self-injuries, but the lesions were less severe and thus they were available to analyze weight and temperature parameters but due to excessive skin lesions, they were not used in locomotor and microdialysis studies. Thus, in total, 122 animals (15 litters) of both sexes with the following genotypes—wild type (WT) (n = 22), DATh (n = 8), DATk (n = 26), D3h (n = 20), D3k (n = 8), DATh/D3h (n = 11), DATk/D3h (n = 19), and DATk/D3k (n = 8)—were used in this study.

### 2.1. Bodyweight

**Figure 1:**
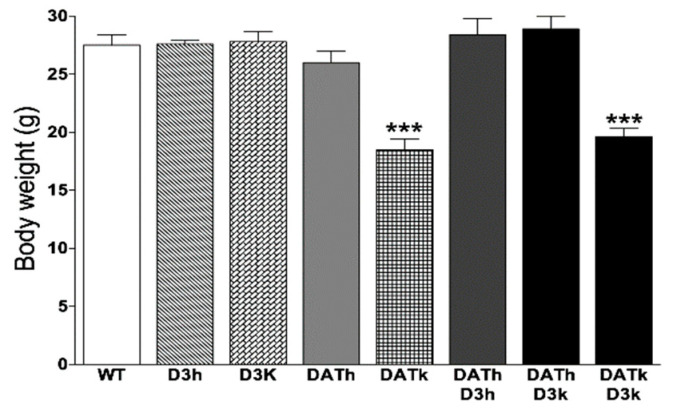
Bodyweight of wild type, dopamine transporter (DAT), and D3 single mutants and DAT/D3 double mutants. Values represented in mean ± S.E.M in grams of body weight. *** *p* < 0.001 vs. wild type (WT) controls.

Body weights were measured in all animals used in these experiments. Consistent with previous observations [2,6], the bodyweight of DATk animals was lower in comparison to WT mice (18.5 ± 0.9 g compared to 27.5 ± 0.9 g, *p* < 0.001). Double knockout mice DATk/D3k also had lower body weight (19.6 ± 0.7 g) compared to WT and D3k animals (*p* < 0.001). Other genotypes did not show any growth abnormalities (DATh—26 ± 1 g, D3h—27.6 ± 0.3 g, D3k—27.8 ± 0.9 g) (Figure 1).

### 2.2. Temperature

**Figure 2:**
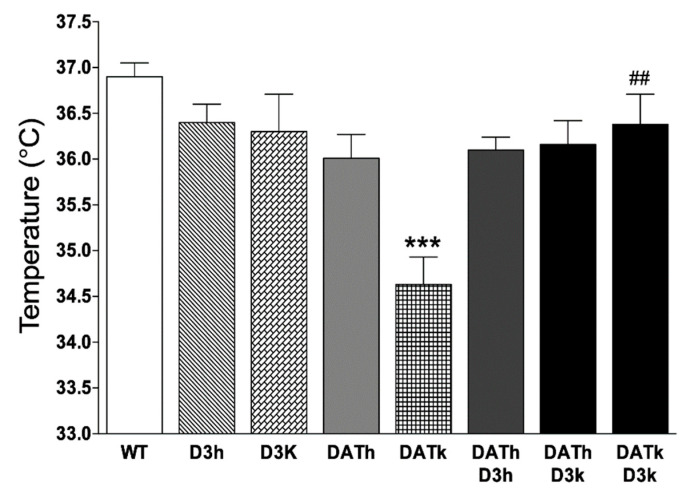
The body temperature of wild type, DAT, and D3 single mutants and DAT/D3 double mutants. Values represented in mean ± S.E.M in °C. *** *p* < 0.001 vs. WT controls, ## *p* < 0.01 vs. DAT knockout (DATk) group.

Body temperature was measured in all animals used in the experiments. Normal body temperature, measured in WT animals, was 36.9 ± 0.2 °C. Mutation in D3 gene did not affect body temperature—both D3k and D3h had normal body temperature, as well as DATh, DATh/D3h, and DATk/D3h (D3k 36.3 ± 0.4 °C; D3h 36.4 ± 0.2 °C; DATh 36 ± 0.3 °C; DATh/D3h 36.1 ± 0.1 °C; DATk/D3h 36.2 ± 0.3 °C). DATk had a significantly lower body temperature 34.6 ± 0.3 °C compared to WT animals (*p* < 0.001). Interestingly, the elimination of the D3 receptor together with DAT protein restored body temperature to WT level (body temperature for DATk/D3k 36.4 ± 0.3 °C) (Figure 2).

### 2.3. Locomotor Activity

**Figure 3:**
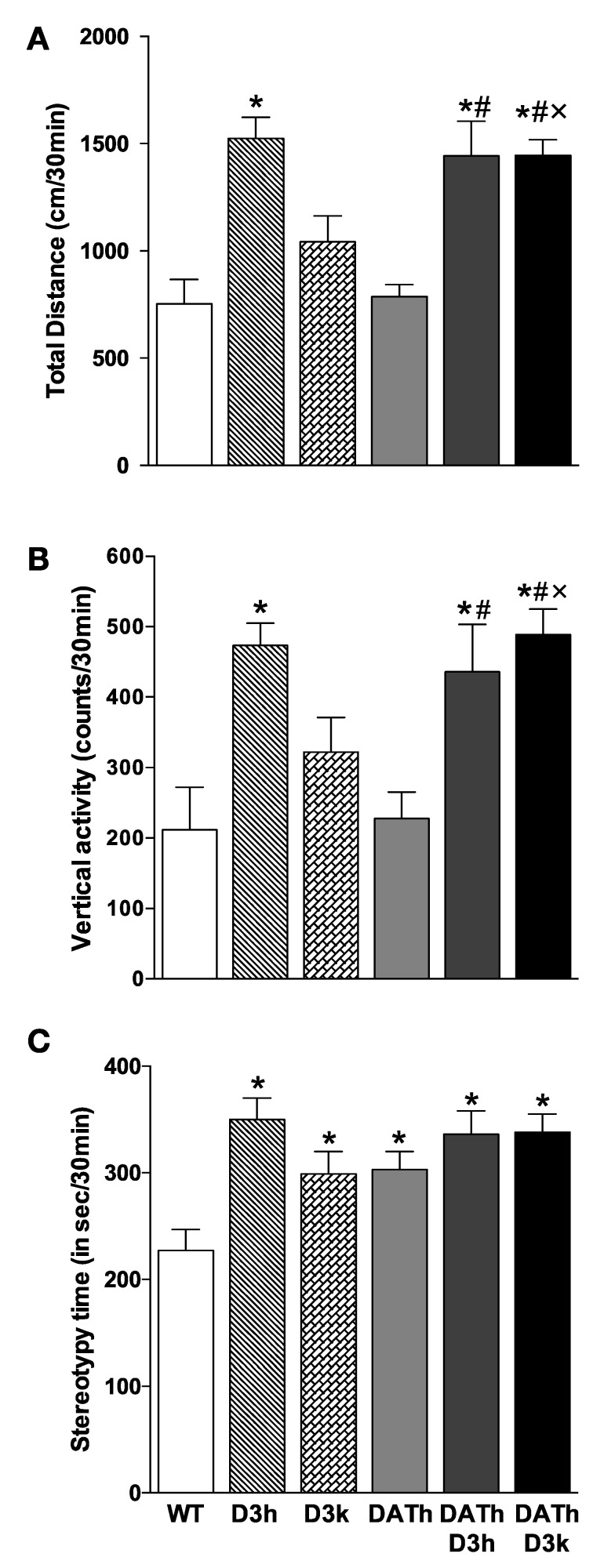
Parameters of locomotor activity of wild type, DAT, and D3 single mutants and DAT/D3 double mutants. Values represented in mean ± S.E.M. (**A**) Total distance shown in cm traveled in 30 min (**B**) vertical activity counts in 30 min (**C**) stereotypy in a total time of stereotypical movement in 30 min. By comparison, the DATk group demonstrated extreme hyperactivity (total distance 8672 ± 1348 cm/30 min; vertical activity 1333 ± 304 counts/30 min; stereotypy time 741 ± 50 s/30 min) with all parameters being significantly different from WT controls (*p* < 0.05). * *p* < 0.05 vs. WT controls; # *p* < 0.05 vs. DATh group; × *p* < 0.05 vs. D3k group.

Spontaneous locomotor activity was measured using an automated monitor system in all animals of all genotypes except for DATk/D3h and DATk/D3k groups. D3k animals had only a slight increase in locomotor activity (Figure 3A). Meanwhile, D3h animals had significantly increased both total distance traveled (Figure 3A) and rearing counts (vertical activity) (Figure 3B) compared to wild type animals (*p* < 0.05).

DATh animals had no pronounced change in locomotor activity, while DAT-KO mice were markedly hyperactive (*p* < 0.05). In heterozygous animals for both genes (DATh/D3h), locomotor activity was elevated compared to DATh animals (*p* < 0.05) but did not exceed the activity of D3h animals. Most interestingly, the activity of DATh/D3k animals was higher compared to both D3k and DATh animals (*p* < 0.05), indicating additive effects of both mutations at the locomotor activity level. Stereotypy score was higher for all mutants compared to WT animals (*p* < 0.05) without significant differences between mutant genotypes (Figure 3C).

### 2.4. Microdialysis Measurements of Striatal Extracellular Dopamine Levels

**Figure 4:**
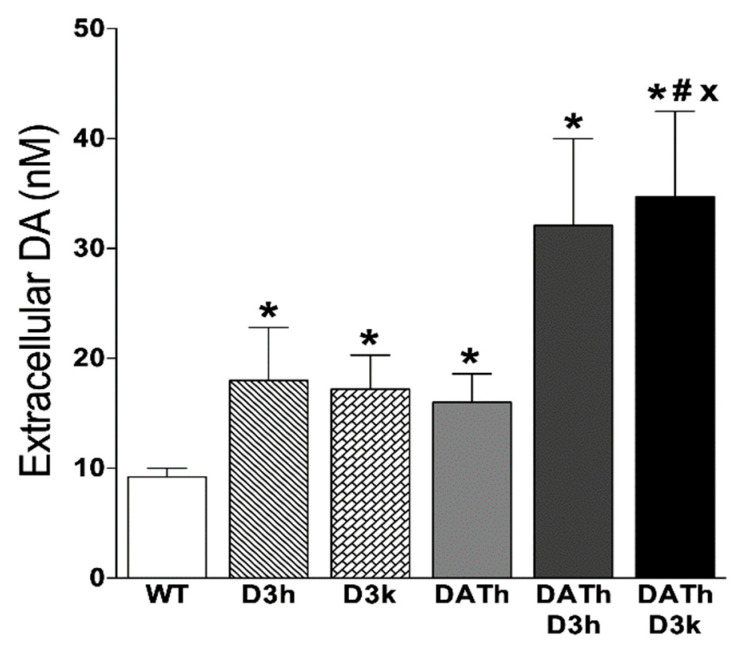
Measures of striatal extracellular dopamine of wild type, DAT, and D3 single mutants and DAT/D3 double mutants detected using quantitative intracerebral microdialysis in freely-moving animals. Values represented in mean ± S.E.M concentration in nM. By comparison, DAT-KO mice demonstrated 48.2 ± 8.1 nM of striatal extracellular dopamine (n = 4) that was significantly different from WT controls (*p* < 0.05). * *p* < 0.05 vs. WT controls; # *p* < 0.05 vs. DATh group; × *p* < 0.05 vs. D3k group.

For microdialysis studies, animals of all genotypes (except DATk/D3h and DATk/D3k) were used: WT (n = 9), DATh (n = 5), DATk (n = 4), D3h (n = 5), D3k (n = 7), DATh/D3h (n = 6), and DATh/D3k (n = 4). The extracellular level of dopamine in the dorsal striatum was measured using a quantitative “low perfusion rate” microdialysis that allows the measurement of “true” extracellular levels of a neurotransmitter [17] (Figure 4). The striatal extracellular level of DA was twice as high in the D3k and D3h animals compared to WT animals (WT 9.2 ± 0.8 nM; D3h 18 ± 4.8 nM; D3k 17.2 ± 3.1 nM) that agree with previous findings in these mutants [13]. The extracellular level of DA for DATh animals was also elevated twice compared to WT (16 ± 2.6 nM) supporting earlier observations [3]. For animals having both of these mutations, the level of extracellular dopamine was even more elevated—in DATh/D3h (32.1 ± 7.9 nM) and DATh/D3k (34.7 ± 7.8 nM) that was about 4 times higher than in WT animals.

## 3. Discussion

In this study, we used double mutant mice with a disruption in either or both DAT and dopamine D3 receptor genes to evaluate the relative contribution of these extracellular dopamine regulatory mechanisms in physiological functions. Furthermore, we aimed to investigate the specific role of the D3 dopamine receptor under the condition of hyperdopaminergia. Dopamine is involved in the regulation of many functions, including movement, motivation, reward, and temperature control [1]. Thus, the dramatic elevation of extracellular dopamine levels due to deficiency in the major regulatory mechanisms can lead to multiple alterations of physiological and behavioral parameters.

The deletion of the DAT gene results in anterior pituitary hypoplasia and dwarfism in mice [6]. DAT elimination dramatically reduces the numbers of lactotrophs and somatotrophs in the pituitary. This effect appears to be related to elevated dopamine levels that down-regulate the lactotroph D2 dopamine receptors and depress hypothalamic growth hormone-releasing hormone content. As a result, DATk mice gain weight slower, and adult animals remain at a lower body weight. Similarly, reduced body weight was also observed in DATk rats [4]. It appears that dopamine function to regulate growth rate represents an evolutionary conservative mechanism. It is known that dopamine can negatively regulate body size in *C. elegance* [18]. However, it seems that not only the level of extracellular dopamine per se could control body growth. Mice lacking D2 dopamine receptors also show growth retardation, although the level of extracellular dopamine is not markedly altered in these mutants [19], so their change in growth is considered to be related to immature growth hormone axis [20]. In contrast, D3k mice have moderately elevated DA levels but it did not affect growth level. In our study, we observed that dwarfism developed in the absence of DAT is observed also in double mutant mice lacking both proteins indicating that the contribution of D3 receptors to this phenotype is minimal.

One of the major neurotransmitters involved in body thermoregulation is dopamine. However, which dopamine receptor is most critically involved in the mediation of the effects on body temperature is still a matter of debate [21,22]. Studies have shown that direct injection of dopamine or its agonist apomorphine into the hypothalamus causes a decrease in core temperature [23]. At the same time, the injection of cocaine, which is known to increase extracellular dopamine levels, does not change basal body temperature but causes impaired heat dissipation [24]. Another example of involvement dopamine in body temperature control is hyperthermia caused by methamphetamine (METH) injection. METH interacts with monoamine transporters, causing elevation of extracellular levels dopamine, serotonin, and norepinephrine. METH-induced hyperthermia is thought to be mediated partially by dopamine. It was shown that in DATk mice METH does not induce an increase in body temperature [25]. At the same time, the DA receptor antagonists protect from METH-induced hyperthermia [26,27]. Injection of D1 receptor-selective agonists is known to cause hypothermia [28]. D1 receptor antagonist SCH23390 did not affect body temperature but was able to prevent METH-induced hyperthermia [29]. D2 dopamine receptor antagonist sulpiride had a similar effect. Injection of D2/D3 agonist 7-OH-DPAT also caused temperature change. However, in D2k mice, this compound caused no change in temperature suggesting that the main role in temperature control plays D2 but not the D3 receptor [30]. It is also shown that chronic injection of D3 antagonist PG01037 decreases body temperature and prevents METH-induced hyperthermia [31]. However, other studies have shown that certain D3 agonists can decrease body temperature, but this effect was also observed in D3k mice, suggesting that these agonists are not selective and their action on body temperature is mediated by D2 receptors [30]. Therefore, the involvement of D3 receptors in temperature control remains questionable. In agreement with previous observations [7], we observed that hyperdopaminergic DATk mice demonstrated lower body temperature. However, in DATk/D3k mice, this decrease in body temperature was abolished. This might suggest that the dopamine level per se is not solely responsible for body temperature changes, and a complex balance of activation of different dopamine receptors is at play. At the same time, it indicates that hypothermia observed in DATk mice is mediated through D3 receptors.

Several mechanisms regulate extracellular dopamine levels. The major one is reuptake of dopamine by DAT [2,3,4,16]. Disruption of DAT expression leads to the persistently elevated extracellular dopamine level due to prolonged time of dopamine clearance after release [2,3,4]. Another mechanism of regulation activity of the dopamine system is autoreceptor-mediated control of dopamine function. Both D2 and D3 receptors are thought to function as autoreceptors. Dopamine autoreceptors can inhibit dopamine synthesis, release, and neuronal firing rate [32,33,34,35]. While the role of D2 dopamine autoreceptors in the regulations of the neuronal firing rate, synthesis, and thus secretion is well accepted, there are several lines of evidence suggesting an additional contribution of D3 receptors in the regulation of dopamine release [33,36].

In agreement with previous studies, we observed elevated quantitative microdialysis measures of extracellular dopamine levels in both D3 and DAT mutant mice [12,37]. Lack of D3 receptors together with partial DAT deficiency in DATh mice resulted in an even higher level of extracellular dopamine. DATk mice are known to have increased D3 receptor gene expression in the striatum indicating an increased level of postsynaptic D3 receptors in response to the persistently elevated extracellular dopamine [8]. At the same time, the elevation of the extracellular level of dopamine due to the lack of DAT results in significant down-regulation of the autoreceptor function to balance the hyperactive tone of dopamine [38]. A higher level of extracellular dopamine in the absence of D3 and partial deficiency of DAT supports the hypothesis that the D3 receptor can be functional as an autoreceptor and compensate to some extent hyperdopaminergic state at least in DATh mice.

Dopamine is critically involved in the control of locomotion. It is well established that an increased level of extracellular dopamine leads to enhanced locomotion [2,3,4,5,16]. At the same time, lack of dopamine leads to akinesia, as it was shown in the dopamine-depleted DATk mouse model [39]. As expected, we observed pronounced hyperactivity of DATk but not DATh mice in our experiments [2,3,4,5]. At the same time, it is well known that D2/D3 dopamine agonists can inhibit the locomotor activity of animals through autoreceptor-related mechanisms [30]. It was reported that inhibitory effects of D2/D3 agonists on locomotion were preserved in D3 knockout mice [30], whereas in D2 knockout mice, these effects were not apparent, suggesting that D2 receptors are more important in mediating these effects on locomotion [40]. In general, studies in D2k animals showed that D2 receptors are crucial for many components of locomotion [19]. At the same time, different D3k strains of mice showed either increased locomotor activity or no change in it [10,11], but an enhanced stereotypy was also observed [13]. Thus, the particularities of the involvement of D3 receptors in the regulation of locomotor activity are still not fully understood. We observed that partial but not full deficiency of the D3 receptor leads to an increase in horizontal and vertical activities that is consistent with previous experiments [10]. In the case of DATh/D3k double mutants, we observed even more pronounced hyperactivity, compared to D3k or DATh, which could be explained by the loss of D3 autoreceptor function and increased level of extracellular dopamine in these mutants. In DAT heterozygous animals, the effect of a 2-fold elevation of extracellular dopamine on physiological functions seems to be compensated during development. Indeed, DATh animals do not show any signs of abnormalities in growth, locomotion, and body temperature [2,3,4,5,16]. However, if it happens together with the loss of D3 dopamine receptor, double-mutant mice show signs of hyperactivity suggesting that these changes are too significant to be compensated.

D3h mice are known to have a greatly reduced D3 binding, but D3 mRNA can still be detected in D3h mice [10]. In our study, we observed that mice heterozygous for the D3 gene display a more severe phenotype than D3 knockout mice. The reason for more pronounced hyperactivity phenotype in D3h mice and self-injuring behavior in DATk/D3h in comparison to D3k counterparts likely relates to both pre- and post-synaptic localization and function of the D3 receptor. While hyperdopaminergia due to deficiency in autoreceptor regulation is already observed in D3h, elevated dopamine still could be detected by remaining D3 receptors in these partial mutants; however, similarly increased extracellular dopamine is not detectable by D3 receptors in D3k mice lacking D3 receptors. Thus, the total loss of D3 on postsynaptic sites may limit the effect of elevated dopamine caused by a deficiency in D3 mediated autoreceptor function.

The excessive grooming resulting in self-injuring was observed in DATk/D3h and, to a lesser extent, in DATk/D3k animals. Increased grooming was previously reported in D3k mice suggesting that it could be related to increased extracellular DA level [14]. Simultaneous deficiency of DAT and D3 dopamine receptors results in a further increase in grooming behavior, up to the state where self-injuring occurs, as we observed in D3h/DATk and D3k/DATk animals. While our locomotor monitor analysis allowed as to evaluate automatic stereotypy scores, these measures are notoriously known to be not precise. Unfortunately, due to difficulty in analyzing eight strains of mice simultaneously at maximally comparable conditions, we were not able to analyze sequential stereotypy of mutant mice more precisely and detailed according to well established methods [41,42]. While we visually observed multiple skin lesions in the most severely affected double knockout mice, in the automatic analysis, we only observed that the stereotypy score was higher for all mutants compared to WT animals without discrimination between genotypes. Interestingly, patients with several neurodevelopmental disorders including Lesch–Nyhan Syndrome demonstrate self-injurious behavior that is believed to be related to altered dopamine function [43]. The potential contribution of DAT and D3 dopamine receptor-related processes in these manifestations might be an interesting direction for future research.

## 4. Materials and Methods

### 4.1. Animals

Double mutant animals were generated by crossing DATk and D3k mutant lines (developed at Duke University, Durham, NC, USA). Parental DATk and D3k strains of mice of mixed C57Bl6 × 129SvJ background were described earlier [13,16]. Animals were housed four or five animals per cage, at room temperature 23 °C, on a 12 h light/dark cycle with ad libitum access to food and water. After achieving at least 4 months of age, adult mice of both sexes were used in the experiments. In these experiments, male and female mice were analyzed separately, and since no significant sex differences were found, the data were combined. All experiments were conducted with an approved protocol from the Duke Institutional Animal Care and Use Committee (A236-04-08, 20.12.2006) and followed the National Institutes of Health guidelines. Before experiments, animals were genotyped to determine their genotype for DAT and D3 gene separately, according to protocols established previously [13,16].

### 4.2. Locomotion

For measurements of locomotor activity and stereotypy, mice were placed in Plexiglas boxes (size 20 × 20 cm) and activity was registered for 30 min. Measurements were performed during a light period between 14:00 and 18:00 with an Omnitech monitor (AccuScan Instruments, Columbus, OH, USA) equipped with infrared photobeam detectors. The horizontal activity was measured as total distance traveled. Stereotypy was determined as the detection of repetitive movement with an interval of less than 1 s. The vertical activity was measured as beam breaks of the vertical sensors placed 7 cm from the floor of the cage [5].

### 4.3. Temperature

Rectal body temperatures were determined by a digital thermometer (Physitemp, Clifton, NJ, USA). The probe was inserted into the rectum of unanesthetized animals in home cages and maintained until the temperature reading had stabilized (typically within 30 s). Measurements were performed once for each tested animal.

### 4.4. In Vivo Microdialysis

Mice were anesthetized with chloral hydrate (400 mg/kg, i.p.) and placed in a stereotaxic frame. Dialysis probes (2 mm membrane length, 0.24 mm outer diameter, Cuprophane, 6-kDa cutoff, CMA-11, CMA/Microdialysis, Solna, Sweden) with CMA-11 guide cannulae were implanted into the right striatum. The stereotaxic coordinates for implantation of microdialysis probes were (in mm): AP 0.0, DV 4.4, ML 2.5 relative to bregma. The placement of the probe was verified by histological examination after the experiments. Following surgery, animals were returned to their home cages with free access to food and water. Twenty-four hours after surgery, the dialysis probe was connected to a syringe pump and perfused at flow rate 1 μL/min with artificial cerebrospinal fluid (CSF; composition in mM: Na^+^ 150; K^+^ 3.0; Ca^2+^ 1.4; PO_4_^3−^ 1.0; Cl^−^ 155; ESA, Bedford, MA, USA) with 0.25 mM ascorbate, pH 7.3. To measure the ”true” extracellular concentration of dopamine, a quantitative “low perfusion rate” microdialysis was performed [13,17]. CSF was perfused at a flow rate of 0.1 μL/min for 6–7 h and collected in a tube containing 2 μL of 0.4 M HClO_4_ every 90 min. Perfusate samples were assayed for dopamine using HPLC-ECD. Monoamines and metabolites were separated on a microbore reverse-phase column (C-18, 5 Wm, 1U150 mm, Unijet, BAS, West Lafayette, IN, USA) with a mobile phase consisting of 0.03 M citrate-phosphate buffer with 2.1 mM octyl sodium sulfate, 0.1 mM EDTA, 10 mM NaCl, and 17% methanol (pH 3.6) at a flow rate of 90 μL/min and detected by a 3-mm glass carbon electrode (Unijet, BAS, West Lafayette, IN, USA) set at +0.8 V. The volume of injection was 5 μL [38].

### 4.5. Statistical Analysis

Data are presented as the mean ± S.E.M. and were analyzed statistically by one-way ANOVA followed by post hoc Tukey test with the use of the Graphpad Prism program (V.5 GraphPad Software, San Diego, CA, USA). Significance at the *p* ≤ 0.05 level and below is reported.

## 5. Conclusions

A comparative analysis of DAT and D3 single mutants and DAT/D3 double mutants revealed some new functions of D3 receptors under conditions of hyperdopaminergia. The deficiency of DAT or D3 results in an elevated level of extracellular dopamine. The deficiency of both of these proteins results in an even higher elevated striatal extracellular dopamine level in DATh/D3k animals. This leads to an increase in the parameters of locomotor activity in double knockouts resulting in self-injuring behavior likely due to an increased level of stereotypy. For the development of dwarfism, the lack of DAT was crucial and the absence of D3 did not influence body weight either in normal animals or DATk mice. The decrease in body temperature observed in DAT knockout mice was abolished when D3 receptors were also removed indicating a specific role of D3 in hyperdopaminergia-related hypothermia in DATk mice.
